# BioFlow: a non-invasive, image-based method to measure speed, pressure and forces inside living cells

**DOI:** 10.1038/s41598-017-09240-y

**Published:** 2017-08-23

**Authors:** Aleix Boquet-Pujadas, Timothée Lecomte, Maria Manich, Roman Thibeaux, Elisabeth Labruyère, Nancy Guillén, Jean-Christophe Olivo-Marin, Alexandre C. Dufour

**Affiliations:** 1 Institut Pasteur, Bioimage Analysis Unit, Paris, France; 2 CNRS UMR3691, Paris, France; 3 Institut Pasteur, Cell Biology of Parasitism Unit, Paris, France; 4 INSERM U786, Paris, France; 5 Institut Pasteur, Leptospirosis Research Unit, New Caledonia; 6 CNRS ERL9195, Paris, France

## Abstract

Cell motility is governed by a complex molecular machinery that converts physico-chemical cues into whole-cell movement. Understanding the underlying biophysical mechanisms requires the ability to measure physical quantities inside the cell in a simple, reproducible and preferably non-invasive manner. To this end, we developed BioFlow, a computational mechano-imaging method and associated software able to extract intracellular measurements including pressure, forces and velocity everywhere inside freely moving cells in two and three dimensions with high spatial resolution in a non-invasive manner. This is achieved by extracting the motion of intracellular material observed using fluorescence microscopy, while simultaneously inferring the parameters of a given theoretical model of the cell interior. We illustrate the power of BioFlow in the context of amoeboid cell migration, by modelling the intracellular actin bulk flow of the parasite *Entamoeba histolytica* using fluid dynamics, and report unique experimental measures that complement and extend both theoretical estimations and invasive experimental measures. Thanks to its flexibility, BioFlow is easily adaptable to other theoretical models of the cell, and alleviates the need for complex or invasive experimental conditions, thus constituting a powerful tool-kit for mechano-biology studies. BioFlow is open-source and freely available via the Icy software.

## Introduction

The ability of cells to define and alter their shape, maintain cell-cell contact, initiate and regulate movement is central to numerous fundamental biological processes including development, microbial infection, immune response, and cancer metastasis^1^. The mechanisms underlying cell shape and motility involve complex molecular machinery that senses and translates both internal and external signals (mechanical and chemical) into physical quantities. At the mechanical level, deciphering how cells deform and migrate requires a better understanding of the biophysical quantities driving intracellular dynamics, including intracellular pressure, stiffness, viscosity and forces^2^. Unfortunately, many of these quantities cannot be measured directly with current methodologies, and are typically estimated using various indirect or invasive experimental approaches^3^. Many such methods operate at the extracellular level, and typically involve interacting with the cell surface. This can be done either actively, e.g. using micro-pipette aspiration^4^, Atomic Force Microscopy^5^ and micro-particle insertion^6^, or passively, e.g. using Traction Force Microscopy, where the cells freely interact with engineered substrates formed either of micro-pillars of known properties^7^ or filled with fluorescent beads^8, 9^. At the intracellular level however, biophysical measurements remain scarce and limited by experimental constraints. Foreign particles can be inserted inside the cell and tracked through video-microscopy in order to characterise intracellular dynamics (Particle Tracking Velocimetry^10, 11^). This technique generally requires controlled manipulation of the particles, which is usually achieved via magnetic^12^ or optical^13^ tweezers. Unfortunately, these methods are highly localised and do not permit global measurements everywhere inside the cell with high spatial resolution. Moreover, foreign particles may compromise cell survival and are not thus suited for long-term experiments. Finally, extending these techniques to 3D environments poses considerable technical challenges and remains an area of active investigation^14^. A non-invasive alternative to these methods lies in Particle Image Velocimetry (PIV), a method to extract the visual flow of information from time-lapse imaging data^15^. PIV has notably been used to characterise cytoplasmic streaming in migrating cells observed via live microscopy^16^. Unfortunately, PIV is only able to extract velocity measures, and suffers from an inherently low spatial resolution. Moreover, it is unable to capture the flow of material leaving or entering the imaging plane in 2D (from above or below), which restricts its applicability.

In addition to experimental techniques, theoretical modelling has also been largely exploited to decipher cell dynamics at the physical and mechanical levels^17–19^. Theoretical models usually describe a specific physicochemical process (or a subset thereof) with high precision, by considering the various constitutive elements of the cytoskeleton, known molecular pathways, and experimental biophysical measurements (most of which are obtained via the aforementioned techniques)^20–22^. Unfortunately, such models are usually tailored specifically to the problem at hand, and are therefore uneasy to adapt or extend to other cell types, or experimental contexts, where cell dynamics may drastically change^23^. Furthermore, the inability to measure biophysical quantities at the intracellular level renders the validation of such models particularly challenging^21, 22, 24^. Recently, the appearance of hybrid approaches exploiting image analysis and computational modelling have shown promising potential in the inference (or validation) of biophysical models using video-microscopy data. For instance, single-cell segmentation and tracking has been used to fit and validate theoretical models of cortical F-actin distribution during cell reorientation^25^. Likewise, cytoplasmic streams estimated using PIV^16^ have been further exploited to estimate the spatial distribution of intracellular shear stress and pressure using Monte-Carlo based computer simulations^26^. An inherent restriction to these approaches however lies in the dependence of the modelling quality on the preliminary image analysis step (and potential limitations thereof). Also, as mentioned earlier, these implementations are tailored specifically to the theoretical model being validated, and cannot easily be applied to other theoretical models, dimensions or cell types.

Here we describe *BioFlow*, a user- and experimenter-friendly mechano-imaging method able to estimate biophysical quantities everywhere inside the cell in a non-invasive manner, in two or three dimensions, using live cell imaging. This is achieved by combining two mathematical techniques in an integrated framework: (i) optical flow^27^, an image processing method that extracts motion information from video sequences and overcomes several limitations of PIV, and (ii) variational data assimilation, a highly-scalable mathematical framework designed to infer the parameters of a given theoretical model based on a limited set of observations (also called *realisations* of the model)^28^. In the present work, the theoretical model is chosen as a fluid dynamics model of the cell interior comprising several quantities of interest (namely velocity, pressure, forces, and out-of-plane flow in 2D), while the realisations of this model are represented by the motion of intracellular material captured via live microscopy. As a result, the proposed method extracts motion information (i.e. intracellular velocity) from a video-microscopy sequence using optical flow, under the constraint of the fluid dynamics model, thus jointly producing estimates of the pressure, forces and out-of-plane flow (in 2D), everywhere inside the cell up to a single-pixel resolution. This computational strategy offers a number of advantages over existing methods: 1) BioFlow is non-invasive and relies exclusively on live microscopy data; 2) BioFlow produces high-resolution measurements everywhere inside the cell in two or three dimensions; 3) BioFlow is independent of the experimental context and thus easily adapts to other theoretical models, biological specimens and imaging techniques; 4) BioFlow is open-source and available as a ready-to-use module for the Icy software^29^.

We illustrate and validate the efficacy of BioFlow in the context of amoeboid cell migration, by studying the trophozoite stage of the unicellular parasite *Entamoeba histolytica* (the causative agent of human amoebiasis^30^), characterised by the emission of local bulges or “blebs” at the cell surface, acto-myosin contraction forces, and cytoplasmic streaming, typical features of amoeboid motility^31^. We show that using a crude approximation of the intracellular material (modelled as a viscous fluid), BioFlow is able to extract intracellular pressure, forces and velocity everywhere inside cells migrating freely on a conventional substrate, and that these quantities corroborate and extend both theoretical and experimental reports. The versatility of the underlying framework confers great potential on this technique for a wide range of biophysical studies, and thus constitutes a powerful addition to the repertoire of bioimage informatics methods for mechano-imaging studies.

## Results

### BioFlow: theoretical framework

Optical flow methods are commonly applied to the kinematic analysis of video sequences^27^. Their purpose is to estimate a displacement field representing the transport of information (here pixel values) between consecutive image pairs. In the context of fluorescence microscopy sequences, the fluorescence emitted by cellular structures is considered as the information that is transported (or advected) by the cell material. The differences in pixel intensities between consecutive images is assumed to be the result of only this advective transport (and not of a change in brightness), and the relation between two consecutive frames can be expressed as

1 $$I(\overrightarrow{x}+\overrightarrow{u}{\rm{\Delta }}t,t+{\rm{\Delta }}t)=I(\overrightarrow{x},t),$$

where *I* is the image signal and $\overrightarrow{u}$ is the estimated velocity field that propagates the information in *I* over a small time interval Δ*t*. Assuming that the displacements in the scene are small and the imaging speed (or frame rate) is fast, one can also write

2 $$\frac{\partial I}{\partial t}+(\overrightarrow{u}\cdot \nabla )I=0.$$

Furthermore, if the the spatio-temporal variations of the velocity field $\overrightarrow{u}$ are sufficiently small Equation 2 can be integrated over a time interval Δ*t* to find a linear relation between the pixel intensities of two consecutive images *I*_1_, *I*_2_ and the displacement field $\overrightarrow{dx}=\overrightarrow{u}{\rm{\Delta }}t$:

3 $$\nabla {I}_{2}\cdot \overrightarrow{dx}+({I}_{2}-{I}_{1})=0.$$

Finding $\overrightarrow{dx}$ is a classical inverse problem, and is usually solved within a variational framework, i.e. by finding the minimum of a so-called cost (or energy) functional of the form

4 $$\{\begin{array}{rcl}J(\overrightarrow{dx}) & = & {J}_{{\rm{data}}}(\overrightarrow{dx})+{J}_{{\rm{reg}}}(\overrightarrow{dx}),\\ {J}_{{\rm{data}}}(\overrightarrow{dx}) & = & {\int }_{{\rm{\Omega }}}{(\nabla {I}_{2}\cdot \overrightarrow{dx}+({I}_{2}-{I}_{1}))}^{2}d{\rm{\Omega }},\\ {J}_{{\rm{reg}}}(\overrightarrow{dx}) & = & \gamma {\int }_{{\rm{\Omega }}}{\Vert \nabla \overrightarrow{dx}\Vert }^{2}d{\rm{\Omega }},\end{array}$$

where Ω represents the spatial domain where *I* is defined. It is worth noting that this formalism makes no assumptions about the dimensionality of the data and thus holds for both 2D and 3D image data. *J*_data_ is called a data attachment term, and describes how well the displacement field matches the intensity changes between the two consecutive images. This term is however not sufficient to find a unique solution for $\overrightarrow{dx}$, notably in the presence of experimental (imaging) noise. To obtain uniqueness, a so-called regularisation term *J*_reg_ (weighted by a non-negative factor *γ*) is generally added to impose a smoothness constraint on the estimated quantities.

A classical limitation of optical flow is that the smoothness constraint imposed on the estimated velocity field is purely arbitrary and does not take the underlying transport mechanism into consideration. Here, we adapt and extend the standard optical flow framework to bioimaging data using the theory of Optimal Control^32^. The general idea is to constrain the estimated displacement by a theoretical model of the observable motion defined by a number of so-called “state-control” variables *c*, which are estimated concomitantly. Formally, the minimisation problem becomes the following:

5 $$(c,\overrightarrow{dx})={\rm{argmin}}({J}_{{\rm{data}}}(\overrightarrow{dx})+{J}_{{\rm{reg}}}(c,\overrightarrow{dx})),\quad {\rm{subject}}\,{\rm{to}}\,A(c,\overrightarrow{dx})=0.$$

In other words, the goal is to match a dynamical observation (the temporally-varying image signal) with a given theoretical model of the intracellular material, whilst jointly estimating its parameters (the biophysical quantities of interest). This strategy offers two major improvements over existing image-based approaches. Firstly, the regularisation term *J*_reg_ can be specifically tailored to the problem at hand, while eliminating the arbitrary smoothness constraint that may not always hold experimentally. Secondly, solving this problem readily provides estimates for the biophysical quantities that minimize the cost function, without the need for additional simulations or model-fitting steps. This data assimilation strategy is particularly appealing since it is independent of the state-control parameters or theoretical model chosen, and can therefore be applied to a wide range of analogous problems in biology.

The proposed framework, able to extract biophysical parameters using optical flow and data assimilation, is coined **BioFlow**, and is straightforward to implement experimentally as it requires only two inputs: 1) time-lapse imaging data of intracellular dynamics (typically obtained by labelling the intracellular material with fluorescence), and 2) a theoretical model of the observable motion. In the remainder of this work we illustrate the use of BioFlow to study amoeboid motility using a fluid dynamics approach, while we stress that this framework is sufficiently generic and flexible to easily adapt to virtually any experimental context, given these two inputs.

### Case study: modelling amoeboid motility using fluid dynamics

We applied BioFlow to study amoeboid motility, taking as a model organism the parasite *Entamoeba histolytica*, the causative agent of human amoebiasis, a disease still today characterised by substantial mortality and morbidity^30^. This unicellular parasite is an appealing model from a biophysical standpoint thanks to its relative simplicity, notably due to the lack of microtubules outside the nucleus^33^ and apparent lack of intermediate filaments^34^. The cytoskeleton is therefore essentially formed of microfilaments, also known as actin filaments. During its obligatory amoeboid migration, the cytoplasmic material flows in the direction of motion, a feature common to many primitive cell types^35, 36^ as well as invasive cancer cells^37^. This mode of migration differs from lamelipodium-based motility where the actin turnover rather exhibits retrograde flow at the leading edge^38^. Amoeboid migration is thought to be driven by self-regulation of intracellular pressure and the emission of blebs at the cell surface^31^, as reported using micro-pipette aspiration experiments^4^ as well as theoretical modelling^21, 24, 39^. Here we ask whether BioFlow can complement these studies by quantitatively characterising the intracellular velocities, pressure and forces driving this flow.

#### Imaging data

**Figure 1:**
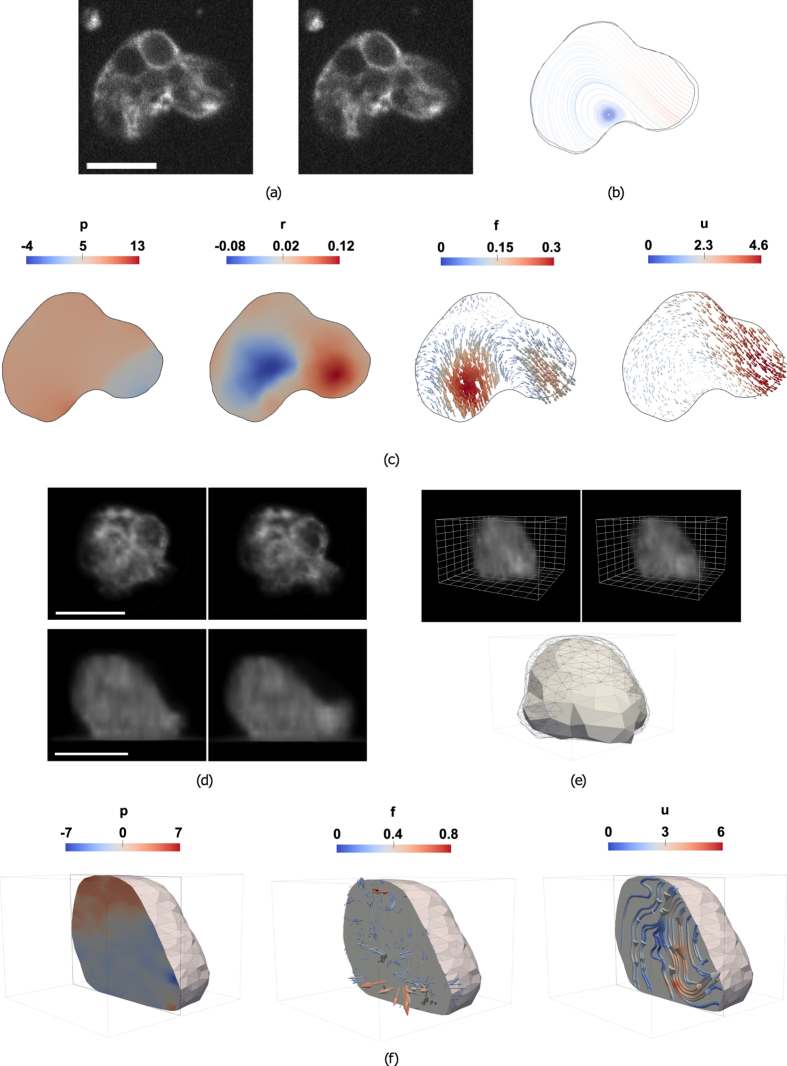
Overview of BioFlow. (**a**–**c**) BioFlow in 2D. (**a**) Two consecutive frames of a 2D time-lapse microscopy sequence (see Supplementary Movie M1 for the full movie); scale bar: 10 *μm*. (**b**) Cell contours extracted from the first (black) and second (grey) frames, and streamlines of the extracted velocity field (integrated using a Runge-Kutta 4–5 algorithm). (**c**) From left to right, estimated 2D intracellular pressure *p* (*Pa*), out-of-plane flow *r* (*s* ^−1^), forces *f* (*nN*/*μm* ^2^) and velocity *u* (*μm*/*s*). (**d**–**f**) BioFlow in 3D. (**d**) Axio-lateral slices of two consecutive frames of a 3D time-lapse microscopy sequence. (**e**) Top row: 3D volume rendering of (**d**); grid spacing 2 *μm*); Bottom row: Cell contours extracted from the first (black wireframe) and second (solid mesh) frames. (**f**) From left to right, sliced view of the estimated 3D intracellular pressure, forces and velocity (the velocity field is displayed as streamlines for better visualisation).

We labelled actin with fluorescent Cytochalasin D (in conditions where the drug concentration does not modify the motility of the cells, see *Methods*) and performed live 2D and 3D fluorescence microscopy experiments (see Fig. 1 and Supplementary Movie S4). Cytochalasin D binds to the free barbed-end of actin filaments^40^, and yields the appearance of short fluorescent filaments in suspension within the cytoplasm. The observable motion of this actin bulk well describes that of the cytoplasm everywhere inside the cell during its movement, and thus constitutes a good input for the algorithm.

#### Theoretical model

We modelled the observable intracellular material as a single-phase homogeneous fluid, neglecting for now the influence of organelles such as the nucleus and intracellular vesicles, following previous work^12^. The dynamics of a single-phase fluid is governed by the Navier-Stokes equations. Two important figures define the regime of solutions for these equations: the Reynolds number ${Re}=\rho VL/\mu$ (*ρ*: density; *V*: velocity; *L*: characteristic distance; *μ*: viscosity) and the Mach number *M* = *c*/*c*_0_ (*c*: characteristic speed; *c*_0_: speed of sound). The Reynolds number is very small in cells (of the order of 10^−5^), implying that the flow is laminar and that both the time-dependent and advection terms in the Navier-Stokes equations can be neglected. In other words, inertia plays no role at the cell scale^41^. The Mach number is small enough such that the fluid can be considered incompressible. Finally, while the cytoplasm is generally considered visco-elastic rather than purely viscous, the elasticity component essentially affects deformations at short (milisecond) time-scales, and therefore has a negligible impact on whole-cell movements at longer (0.1 second) time-scales^42^. Given these assumptions, the cell medium can be considered as a Newtonian fluid governed by the Stokes equations:

6 $$\{\begin{array}{ll}\nabla p-\mu {\nabla }^{2}\overrightarrow{u}=\overrightarrow{f} & {\rm{in}}\,{\rm{\Omega }}\\ \nabla \cdot \overrightarrow{u}=0 & {\rm{in}}\,{\rm{\Omega }}\\ \overrightarrow{u}=\overrightarrow{g} & {\rm{on}}\,{\rm{\Gamma }}\end{array}$$

where $\overrightarrow{f}$ are the local forces (per unit volume) acting on the cell medium and $\overrightarrow{g}$ is the boundary velocity at the cell membrane. The first equation expresses the balance of all the forces acting on the fluid: the first term corresponds to viscous drag (the laplacian acts as a neighbourhood averaging operator on the velocity, i.e. the fluid elements drag each other), the second term is the pressure gradient (propagating local forces throughout the fluid), and the third term accounts for the sum of all other forces, be they internal (e.g. contractility due to myosin activity) or external (e.g. gravity). The second equation indicates that the flow is not divergent, or equivalently that the fluid is incompressible and the mass is conserved. The third equation corresponds to the boundary condition (Dirichlet conditions in the present case). This set of partial differential equations (PDEs) defines the candidate model A in Equation 5. It is worth stressing that the model presented here simplifies the numerical complexity of the problem, yet the proposed methodology is highly generic, and is easily adaptable to any fluid dynamics model of the intracellular material.

#### BioFlow in 3D: general case

Given 3D imaging data and the aforementioned fluid dynamics model, BioFlow is now able to recover the intracellular velocity, pressure and forces from the observable motion of intracellular material by solving the following problem:

7 $$\overrightarrow{u},p,\overrightarrow{f},\overrightarrow{g}={\rm{argmin}}({J}_{{\rm{data}}}(\overrightarrow{u}{\rm{\Delta }}t)+{J}_{{\rm{reg}}}(\overrightarrow{f},\overrightarrow{g})),{\rm{subject}}\,{\rm{to}}\,A(\overrightarrow{u},p,\overrightarrow{f},\overrightarrow{g})=0$$

where

8 $$A(\overrightarrow{u},p,\overrightarrow{f},\overrightarrow{g})=\{\begin{array}{ll}\nabla p-\mu {\nabla }^{2}\overrightarrow{u}-\overrightarrow{f} & {\rm{in}}\,{\rm{\Omega }}\\ \nabla \cdot \overrightarrow{u} & {\rm{in}}\,{\rm{\Omega }}\\ \overrightarrow{u}-\overrightarrow{g} & {\rm{on}}\,{\rm{\Gamma }},\end{array}$$

and the regularisation term rewrites to

9 $${J}_{{\rm{reg}}}(\overrightarrow{f},\overrightarrow{g})=\alpha {\int }_{{\rm{\Omega }}}{\Vert \overrightarrow{f}\Vert }^{2}d{\rm{\Omega }}+\gamma {\oint }_{{\rm{\Gamma }}}{\Vert {\nabla }_{{\rm{\Gamma }}}\overrightarrow{g}\Vert }^{2}d{\rm{\Gamma }},$$

where *α* and *γ* are non-negative empirical weights. In optical control terms, the velocity $\overrightarrow{u}$ and pressure *p* describe the state of the system, and are controlled by the force $\overrightarrow{f}$ and boundary condition $\overrightarrow{g}$ via *A* = 0. Solving this problem numerically (see Material & Methods) therefore produces estimates for these quantities simultaneously in the entire intracellular domain. Again, we stress that this theoretical formulation can accommodate virtually any model for the cellular material, by adjusting the constraint model *A* and the regularisation ${J}_{{\rm{reg}}}(\overrightarrow{f},\overrightarrow{g})$.

#### BioFlow in 2D: handling out-of-plane flow

While BioFlow was designed for the general case of 3D imaging data, we recognise that live cell imaging in 3D may raise technical limitations, either by compromising cell viability due to photo-toxicity, or by not fulfilling the requirement that the effective intracellular motion is small with respect to the observation (imaging) speed. In case 2D imaging is preferred, conservation of mass between consecutive images is no longer a valid assumption, due to the appearance and disappearance of intracellular material above or below the imaging plane of focus. We therefore amended our model in 2D to compensate for this artefact. This is achieved by introducing an additional term *r* that estimates the divergence of the observed flow. Under this condition, the general problem to solve is

10 $$\overrightarrow{u},p,\overrightarrow{f},\overrightarrow{g},r={\rm{argmin}}({J}_{{\rm{data}}}(\overrightarrow{u}{\rm{\Delta }}t)+{J}_{{\rm{reg}}}(\overrightarrow{f},\overrightarrow{g},r)),{\rm{subject}}\,{\rm{to}}\,A(\overrightarrow{u},p,\overrightarrow{f},\overrightarrow{g},r)=0$$

where the candidate model *A* becomes

11 $$A(\overrightarrow{u},p,\overrightarrow{f},\overrightarrow{g},r)=\{\begin{array}{ll}\nabla p-\mu {\nabla }^{2}\overrightarrow{u}-\overrightarrow{f} & {\rm{in}}\,{\rm{\Omega }}\\ \nabla \cdot \overrightarrow{u}-r & {\rm{in}}\,{\rm{\Omega }}\\ \overrightarrow{u}-\overrightarrow{g} & {\rm{on}}\,{\rm{\Gamma }},\end{array}$$

while the functional needs to be regularised accordingly, i.e.:

12 $${J}_{{\rm{reg}}}(\overrightarrow{f},\overrightarrow{g},r)=\alpha {\int }_{{\rm{\Omega }}}{\Vert \overrightarrow{f}\Vert }^{2}d{\rm{\Omega }}+\gamma {\oint }_{{\rm{\Gamma }}}{\Vert {\nabla }_{{\rm{\Gamma }}}\overrightarrow{g}\Vert }^{2}d{\rm{\Gamma }}+\eta {\int }_{{\rm{\Omega }}}{\Vert \nabla r\Vert }^{2}d{\rm{\Omega }},$$

where *η* is a non-negative empirical weight. Note that ${\Vert \nabla r\Vert }^{2}$ could also be replaced by *r* ^2^, should *r* be assumed to be small. This new 2D problem is then numerically solved in the same way as the general 3D case (see *Methods*). It is worth noticing that *r* is a measure of out-of-plane motion. Given that the fluid is considered incompressible, and assuming that fluorescence does not degrade between consecutive images, the 2D motion cannot diverge ($\partial {u}_{x}/\partial x+\partial {u}_{y}/\partial y=0$) and thus $r=-\partial {u}_{z}/\partial z$ reflects the flow in the z-direction. In fact, the volume of fluid leaving the imaging plane between two images is ${\rm{\Delta }}t{\int }_{{\rm{\Omega }}}{u}_{z}\,d{\rm{\Omega }}$, while the change in cell area is ${\rm{\Delta }}t{\int }_{{\rm{\Omega }}}rd{\rm{\Omega }}$. From a Dynamical Systems perspective, the fact that this model does not heavily enforce the conservation of mass permits the creation of velocity sinks and sources, instead of relying exclusively on saddle points. The out-of-plane reformulation of the problem is illustrated in Figure S1 and Supplementary Movie S10.

### Biophysical measurements inside the cell

**Figure 2:**
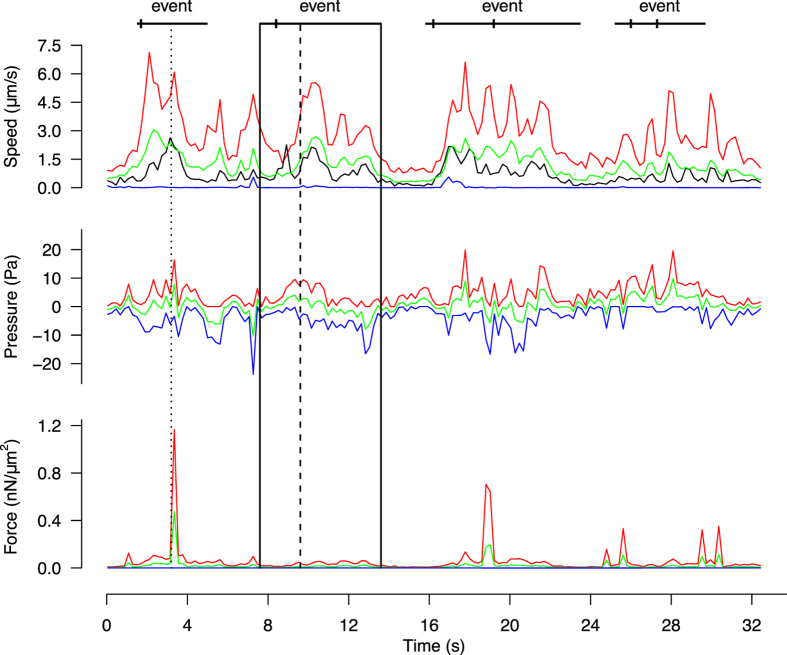
Temporal profile of intracellular quantities during amoeboid migration. Maximum (red line), minimum (blue line) and average (green line) magnitude of the intracellular velocity, pressure and forces measured over the whole cell (cf. Supplementary Movie S4). The black line represents the velocity of the cell centroid, calculated via automated cell tracking. The dotted line (*t* = 3.2 *s*) corresponds to Fig. 1(a). The black rectangle isolates a portion of the sequence surrounding a protrusion event (dashed line at *t* = 9.6 *s*), and is further analysed in Fig. 3.

Figure 1 depicts the biophysical quantities (here velocities, pressure, forces, and out-of-plane flow in 2D) estimated from two consecutive images in 2D and 3D (cf. Supplementary Movies S6 and S7). All quantities are estimated in each node of the underlying Finite Element mesh (see Methods), which can be defined up to a single-pixel resolution (cf. Figure S2). By repeating this analysis over time (cf. Figs 2 and 6(b)), BioFlow enables a rich quantitative analysis of the intracellular dynamics in both space and time. In the remainder of this work we illustrate the results in 2D for easier visualisation, noting that measures obtained on both 2D and 3D datasets were found in good agreement both qualitatively and quantitatively.

The **pressure** field *p* (Fig. 1(c)) exhibits a global gradient in the direction of migration, with higher pressure at the rear of the cell and lower pressure at the cell front. Assuming a constant viscosity of 1 *Pa s*, the pressure values are of the order of 1 to 10 Pascals, which is consistent with both theoretical simulations^39^ and in agreement with Darcy’s law (see *Discussion*).

The **divergence** field *r* (Fig. 1(c)) represents out-of-plane flow. On the depicted image pair (see Supplementary Movies S6 for better visualisation), *r* captures material leaving the plane of focus near the cell rear (blue area, ${r}_{{\rm{avg}}}=-0.01\,{s}^{-1}$), as well as material entering the plane of focus towards the cell front (red area, ${r}_{{\rm{avg}}}=0.05\,{s}^{-1}$). A Fermi order estimation suggests that approximately $0.2\,\mu {m}^{3}$ of material leaves the focal plane at the cell rear, and 0.5 *μm* ^3^ flows into the focal plane at the cell front. It is worth noting that the integral of *r* over the entire cell is positive (5 *μm* ^2^ *s* ^−1^), indicating that the visible cell area increases roughly 1 *μm* ^2^, although this does not imply a change in overall cell volume.

The **force** field $\overrightarrow{f}$ (Fig. 1(c)) is represented as an arrow-field indicating the magnitude and the direction of intracellular forces. The sum of all intracellular forces (with the exception of the viscous drag and the pressure gradient) has a magnitude in the order of the 0.1 to 1 *nN*/*μm* ^2^ and is therefore non-negligible when describing the flow (as a reference, the cell depicted here has an area of 240 *μm* ^2^). While such intracellular force measurements are unique and cannot be directly validated using experimental techniques, the estimated values fall well in range with that obtained using traction-based approaches^43–45^.

The **velocity** field $\overrightarrow{u}$ is represented as streamlines (Fig. 1(b)) and as an arrow field (Fig. 1(c)) indicating the magnitude and direction of movement between the two consecutive frames. The streamline representation offers a good indication of the direction of the flow (Fig. 1(b)), and well depicts cytoplasmic streaming in the direction of migration, as expected for amoeboid migration. The flow also describes a global rotational movement stemming from the rear (where the cell displays a slightly concentric motion, indicating contraction) and ending at the front, where the fastest displacements take place. The instant velocity inside the cell has a magnitude in the order of 1 to 10 *μm*/*s*, and faithfully captures the observed movement of intracellular material. This was confirmed by verifying that the average velocity over the cell ($\propto {\int }_{{\rm{\Omega }}}\Vert \overrightarrow{u}\Vert d{\rm{\Omega }}$) was similar to that of the cell centroid $(\propto \Vert {\int }_{{\rm{\Omega }}}\overrightarrow{u}d{\rm{\Omega }}\Vert )$, as illustrated in Fig. 2.

Figure 2 presents the temporal evolution of the minimum (blue), maximum (red) and average (green) values for each quantity within a cell over time (cf. Supplementary Movie S5). A statistical analysis on 20 cells yielded a maximum velocity, pressure range and force magnitude of 9.2 ± 3.8 *μm*/*s* (mean, s.d.), 21.8 ± 6.3 *Pa*, and 0.52 ± 0.70 *nN*/*μm* ^2^, respectively. The maximum intracellular velocity is up to one order of magnitude larger than the instant speed of the cell centroid (cf. Fig. 2, black curve), which is typical of cytoplasmic streaming.

#### Single protrusion analysis

During amoeboid motility, the process driving bleb nucleation and subsequent cell protrusion is thought to be powered by myosin, which is assumed to regulate hydrostatic pressure within the cell by exerting contractile forces on the actin cortex lying beneath the plasma membrane^4, 31^. Here we asked whether the extracted quantities can give more insight into the underlying mechanism, by studying their spatio-temporal profiles during natural (non-induced) protrusion events.

**Figure 3:**
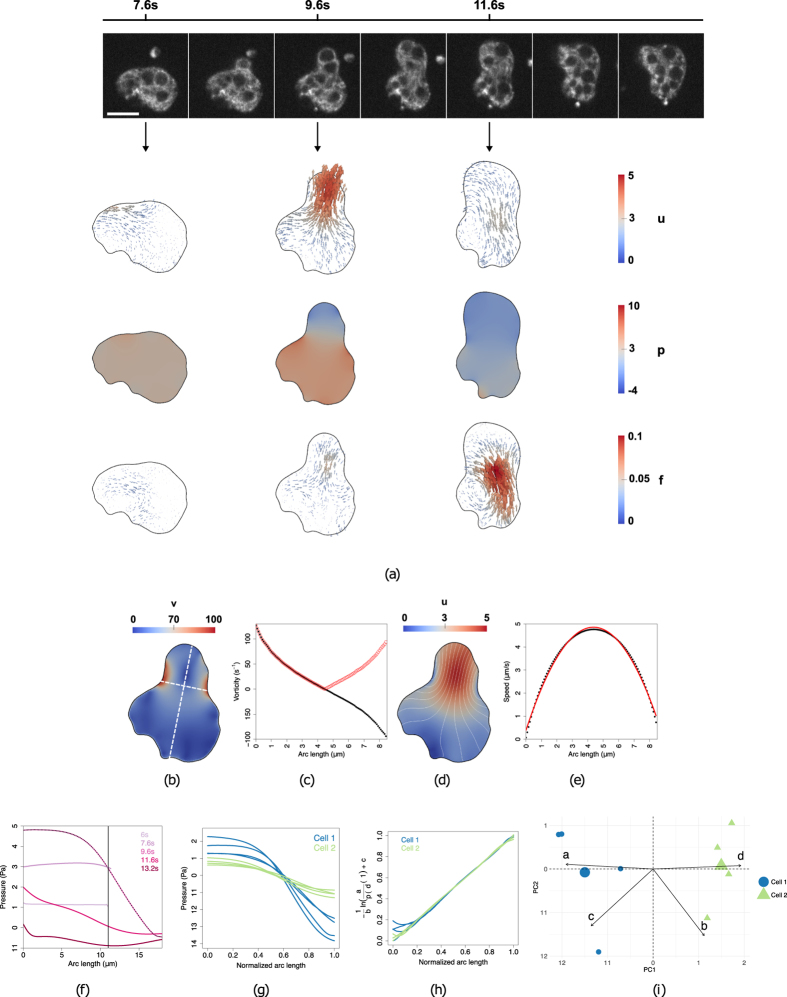
Intracellular velocity, pressure and forces during cell protrusion. (**a**) Top row: snapshots of a 2D video-microscopy sequence (see Supplementary Movie S4); Bottom: estimated intracellular velocity *u* (*μm*/*s*), pressure *p* (Pa) and force *f* (*nN*/*μm* ^2^) before (*t* = 7.6 *s*), during (*t* = 9.6 *s*) and after (*t* = 11.6 *s*) protrusion, respectively. (**b**–**e**) Velocity analysis during protrusion (*t* = 9.6 *s*). (**b**) Magnitude of the vorticity *v* (*s* ^−1^). The long and short white dashed lines indicate cuts along the direction of protrusion and across the bleb, respectively. (**c**) Vorticity profile (black: *v*; red: |*v*|) across the bleb. (**d**) Magnitude of the velocity *u* and its streamlines (white). (**e**) Velocity profile across the bleb (black) and a second-degree polynomial fit (red). (**f**) Pressure values (Y-axis) at several time points along the direction of protrusion (represented on the X-axis from cell rear to cell front); the black line indicates the cell front before blebbing. (**g**) Pressure profiles along the direction of protrusion, obtained on 2 different cells (colour separated) for 4 different protrusion events. (**h**) Sigmoid collapse of the curves in (**g**). (**i**) PCA analysis and k-means clustering of the sigmoid parameters *(a, b, c, d)* (as defined in text) obtained from (**h**). *PC*1 (X-axis) is a linear combination of mainly *a* and *d*, whereas *PC*2 (Y-axis) is a linear combination of mainly *b* and *c*.

Figure 3(a) depicts the biophysical quantities extracted inside the cell before, during, and after protrusion (cf. Fig. 2-black box and Supplementary Movie S5). Before bleb initiation (t = 7.6s) the cell appears stable: the pressure builds up while the velocity and forces remain small. At t = 9.6*s*, a pressure gradient across the cell drives the intracellular material inside the expanding bleb, which is reflected by the increase in velocity at the cell front. After stabilisation of the flow (t = 11.6*s*), the pressure begins to equilibrate, while the cell body moves forward with the help of an increased force.

A detailed analysis of the velocity field during protrusion (Fig. 3(d)) reveals areas where the intracellular material flows from rear to front in a rotating fashion. A vorticity analysis of the velocity field (3(b)) highlights two vortices, one on each side of the bleb, where directions of rotation are opposite. The velocity profile across the bleb (Fig. 3(b), short white line) is smooth and well describes a Poiseuille flow (typical of a viscous fluid flowing through two static plates). This observation was validated by noticing that Poiseuille’s planar equation was able to accurately recover the bleb width (data not shown). This evidence implies that the underlying cortex imposes a no-slip boundary condition to the flow.

Figure 3(f) presents a time-diagram of the intracellular pressure profile measured along the direction of protrusion (Fig. 3(d), long white line). It can be seen that the pressure first builds up from a steady-state, then suddenly drops (suggesting cortex breakage) and creates a decreasing gradient towards the cell front causing intracellular material to flow forward, and stabilises again as the flow stops. Figure 3(g) depicts 4 consecutive pressure profiles extracted over time from 2 different cells. The curves resemble a sigmoid-like shape, which is a characteristic pattern of contraction^46^. Fitting a sigmoid to each curve yields an equation of the form $p=d+a/(1+{e}^{-b(s-c)})$, where *s* is the normalised arc length and (a, b, c, d) are the sigmoid parameters. By transforming the sigmoid into a linear model, i.e. $c-ln(a/(p-d)\,-1)/b$, all curves can be collapsed into the same unitary line (Fig. 3(h)), thereby verifying the sigmoid hypothesis. Strikingly, the parameter space (*a*, *b*, *c*, *d*) is able to capture both inter-cellular and intracellular variations (Fig. 3(i)). The 2 cells can be distinguished using (*a*, *d*). These parameters describe the amplitude range and the height of the sigmoid and thus reflect the ability of each cell to generate pressure. Conversely, (*b*, *c*) are able to distinguish different protrusion events of the same cell. These parameters characterise the spatial geometry of the sigmoid (its slope and position) and therefore reflect the length and position of the protrusion.

**Figure 4:**
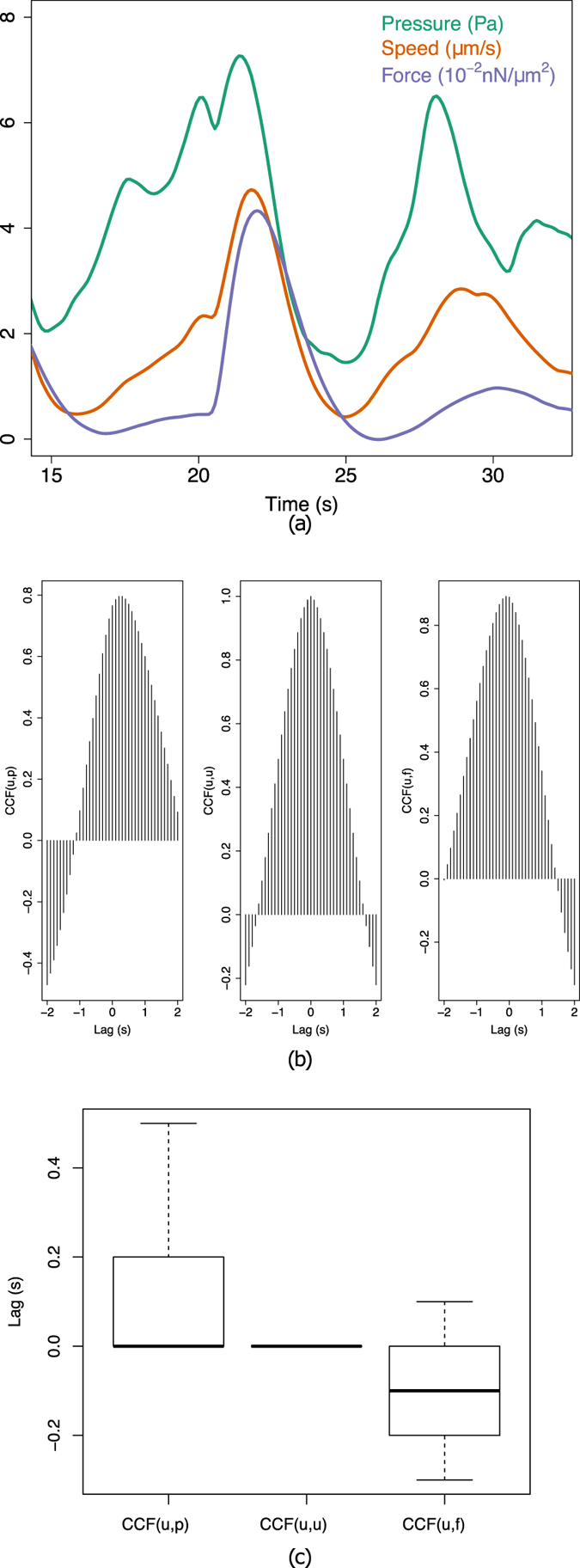
Relative timing of intracellular force, pressure and velocity. (**a**) Estimated range (difference between maximum and minimum across the cell) of the intracellular velocity (red), pressure (green) and force magnitude (blue) for a single cell during a protrusion event. (**b**) Temporal cross-correlation diagrams between *u*, *p* (left), *u*, *u* (middle), and *u*, *f* (right), representing the lag between quantities (given by the shift of the maximum peak away from 0). (**c**) Cumulated analysis of the cross-correlation shift over *n* = 10 cells.

Strikingly, the estimated force field *f* (Fig. 3(a)) seems to have a low overall magnitude during pressure build-up and cytoplasmic streaming, and only increases during the retraction phase, where it localises mostly at the rear of the cell. These observations suggests that the pressure gradient alone is sufficient to initiate cytoplasmic streaming, while cell retraction is not due to the pressure gradient *per se*, but rather hints at a myosin-based mechanism. We further investigated the timing and potential causality between pressure, velocity and forces during protrusion, by performing a correlative analysis over time (Fig. 4). We first plotted the difference between maximum and minimum values (i.e. *range*) for pressure, velocity and forces over the course of a video sequence, and applied a low-pass Butterworth filter^47^ to eliminate spurious small scale fluctuations while preserving the magnitude of the original curve (Fig. 4(a)). We then calculated the pairwise cross-correlation function (CCF) between quantities (Fig. 4(b)) and measured the time lag where the correlation is highest. This analysis was repeated on *n* = 10 cells (Fig. 4(c)) and shows that pressure precedes velocity by 1.1 ± 1.7*s* (mean ± s.d.) and that velocity precedes force by 0.9 ± 1.2*s*, thus further supporting the hypothesis that pressure drives cell expansion while myosin forces drive cell retraction.

#### Periodicity in amoeboid migration

**Figure 5:**
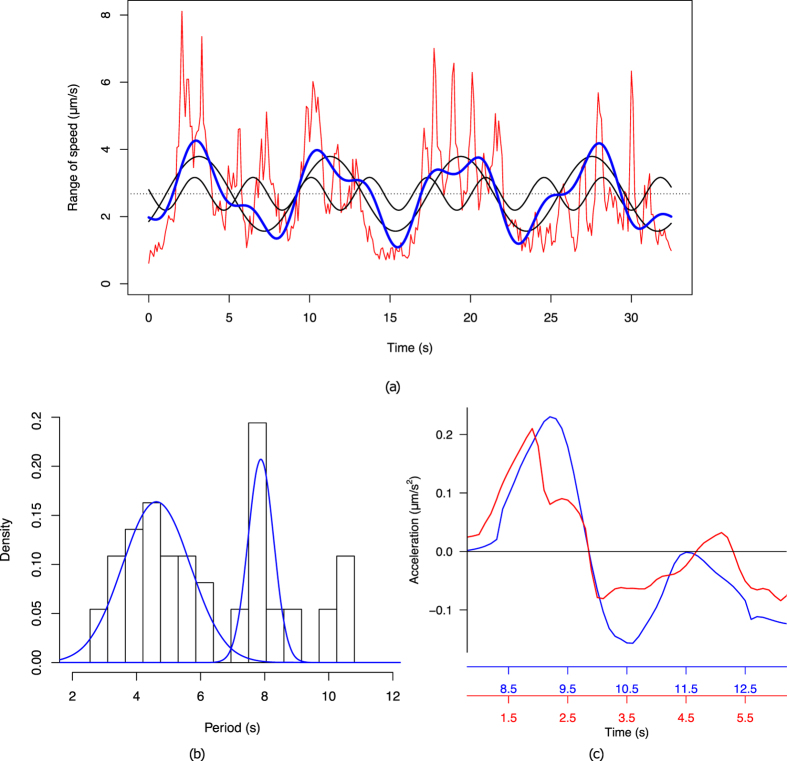
Periodicity in amoeboid migration. (**a**) Velocity range extracted from a single cell over time (red line), and its partial Fourier decomposition (blue line), formed of the average speed (dashed line) and the two most relevant frequency components (black lines). (**b**) Histogram of the associated periods extracted from 10 cells, and the associated sum of Gaussians fit (blue curves) obtained by nonlinear least-squares fitting. (**c**) Time-aligned acceleration profiles along the direction of protrusion for two blebs of a same cell.

A visually striking feature of *Entamoeba histolytica* migration is the apparent periodicity of protrusions (see Figs 2 and 4(a) and Supplementary Movie S4). Previous experiments using micro-pipette aspiration pointed to a periodicity of 8 seconds between protrusion events^4^, but such a measure could not be confirmed in a non-invasive setting. We therefore asked whether BioFlow could allow recovering such a periodicity based on the temporal profiles (Fig. 5). To do so, we analysed the Fourier spectrum of the range of the velocity curve (Fig. 5(a)). Note that a similar analysis could be obtained with the pressure or force profiles, given that all curves are coherent). In this example, the two major cosine functions (in black) that form the experimental measure (in red) have a period of 8.1*s* and 3.6*s* respectively. The blue curve represents the sum of these functions (added to the mean of the original signal), and faithfully reproduces the general patterns of the original signal. We then calculated the density distribution of the two periods by repeating this analysis for *n* = 10 cells (Fig. 5(b)), and obtained two distinct periods of 4.6 ± 1.1*s* (mean ± s.d.) and 7.9 ± 0.4*s* (Non-linear least-squares fitting residual error: 0.03). It is worth pointing out that the longest peak well matches the periodicity obtained using invasive techniques, although it is only visually perceptible in few of the analysed videos (e.g. visible in Supplementary Movie S4, but not in Supplementary Movie S8), thereby demonstrating the robustness of the method.

Most interestingly, the shorter period of 4.6*s*, which is not visually perceptible, describes a more subtle process underlying amoeboid migration. To further highlight this period, we first calculated the average velocity over time across the bleb (i.e. along the line joining the two surrounding vortices, cf. Fig. 3(b)-short white line) and then calculated the corresponding acceleration (i.e. the difference in average velocity between frames). We repeated this analysis on two different protrusion events and superimposed the curves in Fig. 5(c). It can be seen that the intracellular material first accelerates as the pressure gradient establishes, then decelerates as this gradient fades. This cycle takes 3 to 5 seconds, which is well captured by the periodicity analysis, and seems to describe the characteristic time of the cytoplasmic streaming. The higher variance measured for the shorter period may indicate a dependence on protrusion size, which would take longer to fill with cytoplasmic material, given that the streaming velocity is globally homogeneous across cells.

#### Importance of actin dynamics on intracellular flow

**Figure 6:**
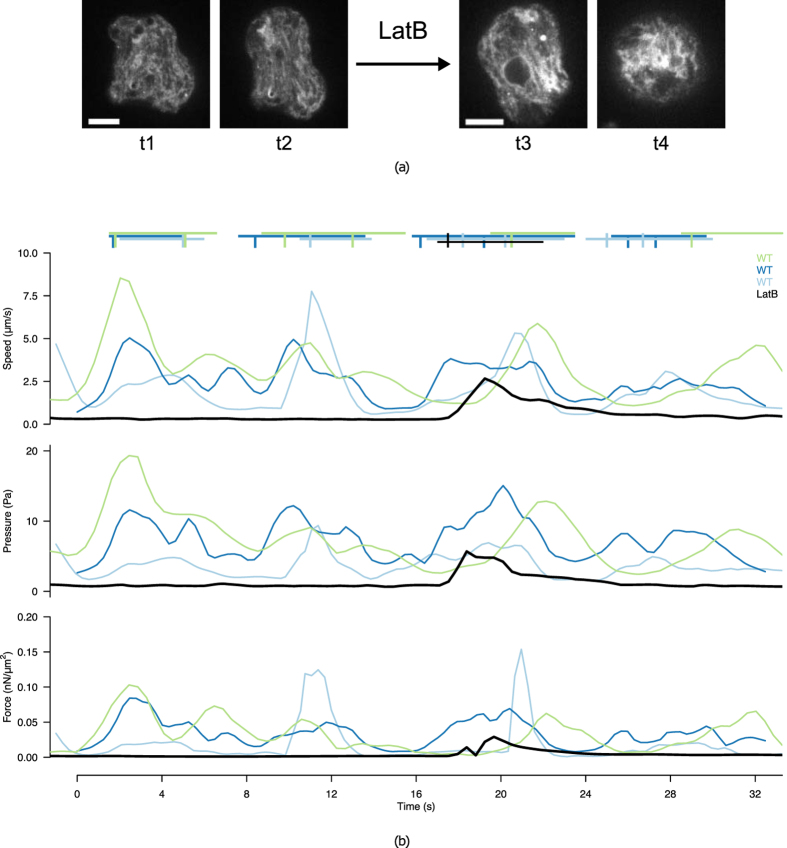
Effect of Latranculin B on cell protrusion. (**a**) Two consecutive frames of a 2D time-lapse microscopy sequence before (left, t1 and t2) and after (right, t3 and t4) addition of Latranculin B. (**b**) Estimated range (difference between maximum and minimum across the cell) of the intracellular velocity, pressure and force magnitude for the treated cell (LatB, black line) and 3 non-treated cells (WT, coloured lines). Protrusion events are marked by segments of the corresponding colour above the graph.

We challenged the experimental model by adding 100 *nM* of Latranculin B in the medium to halt actin polymerisation, and measured the biophysical quantities immediately after the addition of the molecule. The behavior displayed in Fig. 6 is representative of the addition of the drug and contrasts with the behaviour of the wild-type cells. Shortly after addition of Latranculin B (cf. Supplementary Movie S9), the cell exhibits a final protrusion that is remarkably slower than in the control condition (2.5 *μm*/*s* as opposed to 9.2 *μm*/*s* in the control case), and progressively changes from a naturally protrusive phenotype to a rounder and immobile configuration (Fig. 6(a)). This translates quantitatively into a stable decrease in the intracellular pressure gradient, as well as a stabilisation of the velocity and force fields (Fig. 6(b)).

## Discussion

We have developed a powerful and original mechano-imaging framework called BioFlow, which brings together image analysis, physical modelling and mathematical optimisation to perform direct and non-invasive intracellular measurements of invisible mechanical quantities from 2D and 3D fluorescence video-microscopy data. BioFlow was designed as a flexible data assimilation framework, in which we adapted the standard optical flow algorithm such that the observed movement of intracellular material is extracted under the constraints of a biophysical model of the intracellular dynamics. This permits a simultaneous extraction of the intracellular velocity as well as the biophysical parameters of the chosen model, all with high spatial resolution. The flexibility and ease of use of BioFlow lies in three complementary aspects: 1) its theoretical foundation provides a method that is virtually independent of (and adaptable to) any theoretical model of the cell; 2) it relies exclusively on conventional video-microscopy data of living cells, and thus permits non-invasive single cell studies in diverse experimental contexts; 3) its open-source software implementation lets any component of the workflow be tailored to the specific problem at hand (for instance, implementing a new theoretical model solely requires deriving its weak formulation, see Supplementary Information). In this work we presented one possible implementation of BioFlow, where we used a Newtonian fluid dynamics model of the cell material to study the amoeboid motility of *Entamoeba histolytica* trophozoites, and were able to provide high-resolution maps of intracellular velocities, pressure and for the first time, forces and out-of-plane flow (in 2D). While validating some of these measurements is experimentally challenging, we believe that the proposed implementation constitutes a good case study to validate the method, and possibly enrich the landscape of experimental and theoretical reports on amoeboid motility with new quantitative insight.

A hallmark of amoeboid motility is the emission of protrusions at the cell surface, largely believed to be induced (directly or indirectly) by pressure-induced actomyosin contractility^4, 21, 22, 31^. Once a bleb is initiated, the membrane locally delaminates from the cortex and fills with cytosol. In some cases, the bleb retracts without generating movement, while in other cases the actomyosin cortex locally disrupts (either via depolymerisation or breakage), causing an influx of intracellular material into the protrusion. While *Entamoeba histolytica* exhibits both types of behaviour, the Cytochalasin-D labelling used in this work only captures the latter, as it highlights the intracellular actin bulk and not the outer cell membrane. If we consider the pressure drop Δ*p* across the cortex (of thickness *h* ~ 1 *μm* ^20^) necessary to drive the percolation of cytosolic fluid (of viscosity close to water, i.e. *v* ~ 10^−3^ *Pa s*) through the pores of the actin mesh (of characteristic size $l \sim 0.01-0.1\,\mu m$ ^48^), a dimensional analysis leads to a value of ${\rm{\Delta }}p\propto u\nu h/{l}^{2} \sim 1-10\,Pa$. The same relation can also be reached by rearranging Darcy’s law ($u/A\propto \nabla p/\nu$), governing the flow of slow fluids through porous media. Once the actin cortex disrupts, this same pressure gradient then drives the cytoplasm (with a viscosity of *μ* ~ 1 *Pa s* ^12^) inside the bleb. Strikingly, the estimated spatiotemporal profile of the intracellular pressure during protrusion (cf. Fig. 3) estimated by BioFlow is in good agreement with this picture both qualitatively and quantitatively, further supporting the reported role of myosin II: 1) increase in intracellular pressure caused by contraction of the actomyosin cortex; 2) sigmoid-shaped pressure curves during cytoplasmic streaming into the bleb (analogous to a contracting heart, also powered by myosin), with pressure values well matching Darcy’s law estimation; 3) retraction of the cell rear after stabilisation of pressure and accompanied by an increased force. Interestingly, the establishment of the pressure gradient at the onset of protrusion takes only a small fraction of the characteristic time of actin depolymerisation^20^, pointing at a sudden disruption of the cortex. Likewise, pressure stabilisation as the bleb fills is also relatively fast^20^ and therefore suggests a process that is not solely based on repolymerisation. Furthermore, an analysis on multiple protrusion events revealed that absolute pressure values differ across blebs, yet without disturbing periodicity (cf. Fig. 5), indicating that pressure alone does not suffice to regulate bleb formation and stabilisation. This was confirmed by recent evidence that bleb formation and regulation involves additional mechanisms including water exchange at the cell membrane^21^, as well as Rho-GTPase activity^49, 50^.

The velocity field extracted by BioFlow well corroborates existing descriptions of cytoplasmic streaming in other amoeboid cells (Fig. 3(d)). However, our high-resolution measurements, in conjunction with the other estimated quantities, provide further speculative insight into the underlying mechanisms. Indeed, our temporal analysis (Fig. 4) indicates that the pressure gradient precedes the velocity increase of the intracellular material, suggesting a cause-consequence relationship. As the pressure stabilises and the protrusion is filled, the initial acceleration vanishes (cf. Fig. 5(c)). Interestingly, the intracellular material flows towards the protrusion in a rotating fashion, as shown by the two vortices on each side of the bleb (cf. Fig. 3(b)), while the Poiseuille nature of the observed flow (cf. Fig. 3(e)) implies a no-slip boundary condition. This evidence further supports a localised breakage of the cortex, while the remainder of the cortex holds in place. It is also worth pointing out that the cytoplasmic streaming is noticeably smooth (the intracellular material, including diverse vesicles and organelles such as the nucleus, seems to be dragged uniformly and passively), although this was not imposed by the theoretical model. This behaviour might reflect the lack of microtubules in *Entamoeba histolytica* as opposed to other cell types such as *Physarum* amoebae, where the streaming appears more irregular^16^.  Using BioFlow we were able to provide for the first time a detailed map of intracellular forces. Regions of high force magnitude were found to generally appear in one of two cases: (a) when the cell reorients, causing rotational movements (similarly to a Taylor-Couette flow) that might stem from forces exerted by the cell on the substrate (Fig. 1(c) and Movie S5); (b) more frequently, during protrusion events, yet with a slight delay after stabilisation of the pressure gradient and velocity field (Figs 3(a) and 4). This suggests that forces are highest during the retraction phase (i.e. when the bleb is practically filled and the flow stabilises) and might be a consequence of the actomyosin contractility. This hypothesis is further supported by the acceleration profile of the protrusion (Fig. 5(c)), where the first acceleration-deceleration cycle (linked to the pressure drop and stabilisation discussed above) is followed by a second, more subtle acceleration phase concomitant with the force increase.

The robustness of BioFlow is illustrated by the overall coherence of the quantities extracted from multiple cells, and successfully challenged by measuring the dramatic impact of Latranculin B (a known inhibitor of microfilament formation) on the intracellular measures (cf. Fig. 6). This has led us to highlight a characteristic period of 7.9*s* between two consecutive protrusions with low variance (cf. Fig. 5), which is well in line with previous reports using micropipette aspiration^4^, although it had never been measured in free living *Entamoeba histolytica* trophozoites. Furthermore, we were able to isolate a novel, second characteristic period of 4.6*s*, although with a higher variance. Interestingly, we found that this duration matches that of the acceleration-deceleration cycle observed during protrusion (cf. Fig. 5(c)). Since this cycle characterises the cytoplasmic streaming towards the bleb, we speculate that this period could describe the characteristic time of actin cortex re-polymerisation at the edge of the protrusion.

Taken together, our results were found in excellent agreement with the currently accepted model of bleb-based amoeboid motility, and illustrates how BioFlow can be used to either validate or provide novel quantitative insight into the underlying mechanisms. It is worth stressing that in the present work, our choice of theoretical model solely assumes that the observable intracellular material behaves as a fluid, without making any assumption on the molecular mechanisms underlying the observed motion. The interpretation of the estimated quantities therefore depends on the validity of the chosen model. It can be argued, for instance, that the chosen Newtonian fluid model is incomplete and should account for a viscoelastic component^12^. Concretely, this would require replacing the homogeneous viscous stress of the Stokes system (of the form $\mu {\nabla }^{2}\overrightarrow{u}$, cf. Equation 6) by the non-homogeneous form of the full Navier-Stokes system, i.e. $\nabla \cdot (\mu (\nabla \overrightarrow{u}+{\nabla }^{T}\overrightarrow{u})+\tau )$, where the elastic stress *τ* is modelled by an additional evolution equation. Treating *μ* as an additional unknown to the problem is possible, but would substantially increase the complexity of the theoretical model and its numerical resolution. Given the size of the cell and the time-scale of imaging (of the order of 0.1 s), we believe that the uniformity of *μ* is a good preliminary approximation and that the estimated quantities are only minimally affected by the elasticity component. Also, the membrane forces (e.g. pulling, pushing or friction) are not directly accounted for by the chosen physical model, and are implicitly taken into account through the boundary displacements. Yet, the relatively small transversal velocity at the boundary suggests that there is friction on the membrane. Finally, the biophysical model should also be extended to consider the fact that several cellular organelles (such as the nucleus) do not necessarily behave like a fluid, and therefore require a specific biophysical model. In addition to permitting a more faithful estimation of biophysical quantities, this would allow a better segregation of the various forces acting within the cell.

While we believe that BioFlow constitutes a solid and necessary addition to the landscape of methods to study intracellular dynamics in evermore complex environments, we foresee that the power and flexibility of the underlying framework will rapidly broaden the applicability of BioFlow to other fields of life sciences. For instance, the current implementation of BioFlow offers a straightforward and appealing alternative to multi-cellular modelling in developmental biology, where an equivalent Newtonian assumption was used to infer physical quantities from tissue dynamics during drosophila gastrulation^51^. At an even higher scale, the ability to measure out-of-plane flow can be of great interest to decipher the mechanisms establishing and regulating blood-flow in the beating heart, where live imaging in 3D remains a technical challenge^52^.

## Methods

### Cell culture and fluorescent labeling

*Entamoeba histolytica* trophozoites (strain HM1: IMSS) were cultivated axenically in a TYI-S-33 medium^53^ at 37 °C, and incubated for 15 minutes with a fluorescent conjugate of the actin binding drug Cytochalasin D^54^ (Cytochalasin D, Bodipy-TMR FL conjugate) at non-lethal concentration (50 nM, following previous work^55^). Cytochalasin D at this low concentration provides strong fluorescence labeling of free-floating actin polymers, yet without interfering with cytoskeletal functions. This was carefully controlled by verifying that cell morphology and speed did not change at concentrations ranging from 1 to 100 *nM* of Cytochalasin D (median speed: 11 microns per minute for untreated cells vs. 12 microns per minute for labeled cells, measured as previously described^56^). We also controlled the specificity of the fluorescent reporter by adding unlabelled Cytochalasin D, which reduced the signal, indicating that the labelled and unlabelled forms of the drug bind to the same site of F-actin.

### Live cell imaging

Live cell imaging experiments were conducted on an inverted microscope (Axiovert 200 M; Carl Zeiss) equipped with a spinning-disk confocal system (Ultraview ERS, Perkin Elmer) with a 63x oil-immersed objective (plan-Apochromat; 1.4-NA), with a pixel size of 0.16 *μm*. The fluorescent signal (emission wavelength: 545 nm; detection bandwidth: 480-570 nm) was recorded on an electron-multiplying CCD camera (Andor EMCCD DV885), with an exposure time between 50 ms and 100 ms per plane, adjusting the compromise between signal-to-noise ratio and temporal resolution. 3D acquisitions were obtained on the same system by acquiring 12 planes per Z-stack, spaced 0.82 *μm* apart. To increase imaging throughput in 3D, a 2× binning was used (yielding a 0.32 *μm* pixel size) and a rectangular region of interest was defined around the cell of interest to reduce camera readout time. Using these settings, a 3D stack of size 25 × 25 × 10 microns was acquired in 0.663 seconds. Time-lapse sequences in 2D and 3D were acquired at full imaging rate (i.e. no delay between individual time points).

### Numerical and software implementation

Equations 7 and 10 were solved within a variational data assimilation framework^57^ using the Finite Element Method. An iterative gradient descent strategy was chosen over the classical analytical (augmented Lagrangian) approach due to the complexity of the problem, while convergence is guaranteed given that the functional is convex and the problem well-posed. Furthermore, this strategy scales particularly well with the number of variables in the problem, thanks to the use of the adjoint method, which inexpensively computes the gradient of the functional. Multi-scale analysis was implemented to speed up computations and handle potentially large displacements between image pairs. Finally, an automated parameter estimation procedure was implemented to optimise the empirical parameters *α*, *γ* and *η* (Equations 9 and 12). Full details of the numerical implementation are available in Supplementary information.

The final workflow was implemented within the Icy^29^ software and is available as a downloadable module (more details at http://icy.bioimageanalysis.org/plugin/BioFlow). After loading a video-microscopy sequence into the software, an initial contour is defined around the cell on the first frame (this manual step can be automated using any available segmentation technique). The main BioFlow script (written in Jython) is launched from Icy’s script editor and starts by segmenting and tracking the cell boundary over time using an active contour method^58^, then creates a multi-scale version of the image and contour for each frame. The contour-image pairs are subsequently transferred to a python script running the FEniCS^59^ Finite Element software, where the remainder of the computations take place: (a) the image and contour are converted into a Finite Element mesh using the *Mshr* (mesh generation) module; (b) the PDE system in 3D (equation 9) or 2D (11) is then solved using the *MUMPS* solver^60^; (c) finally, the functional *J* is minimised using the *dolfin-adjoint* module. This process is iterated until convergence and repeated from coarse to fine image scales using appropriate warping. To reduce computation time, each pair of frames in the video sequence is processed in parallel using all available processors on the workstation. The final results and figures were produced either within Icy or with the ParaView software (http://www.paraview.org).

## Electronic supplementary material

Supplementary Information

Movie S4

Movie S5

Movie S6

Movie S7

Movie S8

Movie S9

Movie S10
