# Supplementary material for: BioFlow: a non-invasive, image-based method to measure speed, pressure and forces inside living cells
Source: Sci Rep. 2017 Aug 23;7:9178. doi: 10.1038/s41598-017-09240-y (PMC5569094; doi:10.1038/s41598-017-09240-y)
Supplement: Supplementary file 1 — Supplementary Information [file 41598_2017_9240_MOESM1_ESM.pdf]

BioFlow: a non-invasive, image-based method to measure speed, pressure and forces inside living cells

Aleix Boquet-Pujadas[1,2+], Timothée Lecomte[1,2+], Maria Manich[1,2], Roman Thibeaux[3,4,5], Elisabeth Labruyère[1,2], Nancy Guillén[3,4,6,7], Jean-Christophe Olivo-Marin[1,2*], and Alexandre C. Dufour[1,2*]

[1]Institut Pasteur, Bioimage Analysis Unit, Paris, France
[2]CNRS UMR3691, Paris, France
[3]Institut Pasteur, Cell Biology of Parasitism Unit, Paris, France
[4]INSERM U786, Paris, France
[5]Current address: Institut Pasteur, Leptospirosis Research Unit, New Caledonia
[6]Current address: CNRS ERL9195, Paris, France
[7]Current address: INSERM U1201, Paris, France
[+]These authors contributed equally to this work
[*]Correspondence: adufour@pasteur.fr, jcolivo@pasteur.fr

ABSTRACT

Cell motility is governed by a complex molecular machinery that converts physico-chemical cues into whole-cell movement. Understanding the underlying biophysical mechanisms requires the ability to measure physical quantities inside the cell in a simple, reproducible and preferably non-invasive manner. To this end, we developed BioFlow, a computational mechano-imaging method and associated software able to extract intracellular measurements including pressure, forces and velocity everywhere inside freely moving cells in two and three dimensions with high spatial resolution in a non-invasive manner. This is achieved by extracting the motion of intracellular material observed using fluorescence microscopy, while simultaneously inferring the parameters of a given theoretical model of the cell interior. We illustrate the power of BioFlow in the context of amoeboid cell migration, by modelling the intracellular actin bulk flow of the parasite *Entamoeba histolytica* using fluid dynamics, and report unique experimental measures that complement and extend both theoretical estimations and invasive experimental measures. Thanks to its flexibility, BioFlow is easily adaptable to other theoretical models of the cell, and alleviates the need for complex or invasive experimental conditions, thus constituting a powerful tool-kit for mechano-biology studies. BioFlow is open-source and freely available via the Icy software.

Supplementary information

Numerical resolution using data assimilation and the Finite Element method

Here, solving the problem defined by Equation 5 involves minimising a functional (Equations 9 or 12) constrained by a system of partial differential equations (PDE) with non-zero boundary conditions (Equations 9 or 11 with $A = 0$). The minimisation process provides an estimate for three quantities, namely the boundary velocity $\vec{g}$, the force field $\vec{f}$ and the divergence r in 2D (we hereafter refer to this set as $m = (\vec{g}, \vec{f}, r)$), while the velocity $\vec{u}$ and pressure p are obtained by solving the PDE for a given m (we let hereafter $\theta(m) = (\vec{u}, p)$). In the field of mathematical optimisation, such a constrained minimisation problem is known to be particularly complex to solve numerically. A classical solution is to transform the problem into an unconstrained optimisation framework by introducing Lagrange multipliers[1]. Unfortunately, this strategy does not scale well when the number of unknowns is large. Here m contains two vector fields and a scalar, defined on a grid with a size depending on the spatial resolution of the acquired images, which is potentially large. In such situations, the standard transformation with Lagrange multipliers, although theoretically possible, is not computationally tractable.

Here we adopt an alternative strategy, namely iterative gradient descent, which is numerically simpler and theoretically guarantees convergence because the functional is convex and well-posed (m is regularised). Implementing gradient descent comprises two main steps, which are repeated sequentially until convergence: 1) computing the functional J (which requires solving the PDE system for a given m), and 2) computing the gradient of J, which provides a direction in the parameter space m where a better solution can be found.

Step 1: computing J

The first step requires solving the so-called *forward problem* defined in equation 11 with $A = 0$: calculate $\theta = (\vec{u}, p)$ given $m = (\vec{g}, \vec{f}, r)$.

To avoid potential numerical round-off errors due to working with both small and large numbers, and to assess the importance of each term in the equations, we rewrite the system into a dimensionless form by posing the following change of variables: $x = x^* l$, $y = y^* l$, $p = p^* \frac{\mu}{\Delta t}$, $\vec{f} = \vec{f}^* \frac{\mu}{\Delta t l}$, $\vec{u} = \vec{u}^* \frac{l}{\Delta t}$, $r = r^* \frac{1}{\Delta t}$, $g = g^* \frac{l}{\Delta t}$. Furthermore, to simplify the resolution, we modify the problem such that it has zero-boundary conditions, by posing $\vec{u}^* = \vec{u} + \vec{u}_0$. With these new variables (and dropping the asterisk symbol for easier reading), equation 11 becomes:

$$\begin{cases} \nabla p - \nabla^2 \vec{u} &= \vec{f} + \nabla^2 \vec{u}_0 & \text{in } \Omega \\ \nabla \vec{u} &= r - \nabla \vec{u}_0 & \text{in } \Omega \\ \vec{u} &= 0 & \text{on } \Gamma \\ \vec{u}_0 &= \vec{g} & \text{on } \Gamma \end{cases} \tag{13}$$

Another possibility to deal with the boundary condition would be to apply Nitsche's method. In any case, equation 13 cannot be solved analytically. We therefore relax the problem by deriving its so-called *weak variational form*. The idea is to integrate the first (momentum) equation and the second (conservation) equation over the domain of interest, weighted by some test functions $\vec{v}$, q:

$$\begin{aligned} w &= a(\vec{u}, \vec{v}) + b(\vec{v}, p) + b(\vec{u}, q) \\ &= \int \nabla \vec{u} \cdot \nabla \vec{v} \, d\Omega + \int p \nabla \cdot \vec{v} \, d\Omega + \int q \nabla \cdot \vec{u} \, d\Omega, \\ L &= -\int \nabla \vec{u}_0 \cdot \nabla \vec{v} \, d\Omega + \int \vec{f} \cdot \vec{v} \, d\Omega - \int q \nabla \cdot \vec{u}_0 \, d\Omega + \int q r \, d\Omega, \end{aligned} \tag{14}$$

where the pressure sign is switched for stability. Our goal is now to find a pair of trial functions velocity and pressure $(\vec{u}, p)$ belonging to some mixed space $V \times M = {H_0}^1(\Omega)^n \times {L_0}^2(\Omega)$ such that $w = L$ for all test functions $\vec{v}$ and q belonging to $V \times M$. While this reformulation is valid (in the sense of distributions), solutions may exist without necessarily satisfying the original system of equations. This is however a reasonable simplification, given that many laws of physics expressed in terms of differential equations are often derived from their original integral form. For example, the incompressibility equation $\nabla \cdot \vec{u} = 0$ is derived from the continuity equation $\int \nabla \cdot \vec{u} \, d\Omega = 0$ originally expressing the conservation of mass (and recovered in equation 14). Furthermore, given that here a is a continuous coercive bilinear form and b is a continuous bilinear form satisfying the LBB condition, the Brezzi splitting theorem[2] states that, given a reasonable $\vec{f}$, a unique solution to this problem exists.

To solve this new problem numerically, we take advantage that the weak mathematical formulation is compatible with the **Finite Element Method** (FEM), a popular method in the engineering and computational modelling community. Applying FEM consists in dividing the domain Ω in smaller subdomains that are well approximated by simple equations, such that the problem can be solved independently on each subdomain, while a global solution to the weak-formulation problem can be reassembled from the local solutions. This is achieved here by discretising the function space, by representing our trial and test functions using a basis of, piecewise polynomials. Piecewise functions guarantee that subdomains are independent, while polynomials provide a good local approximation. In our case, the stability of the solutions of the Stokes equations is ensured using a specific combination of these finite elements[3]. Here we use Taylor-Hood elements[4], where the velocity and the pressure are respectively described as piecewise quadratic and linear polynomials, ensuring that the discretisation of the weak form is stable and well-posed. Finally, by substituting the discretised functions in the weak formulation (Equation 14), the problem reduces to solving a linear system of equations.

Step 2: computing the gradient of J

The second step involves computing the gradient of J with respect to the control functions (dJ/dm, i.e. $dJ/d\vec{g}$, $dJ/d\vec{f}$ and dJ/dr), which is computationally complex, notably for high-dimensional problems. A classical finite difference approach would be computationally intractable, since it involves computing J (step 1) several times for each degree of freedom in m. Instead, we use the adjoint method[5]. The idea is to apply the chain rule to $J(\theta(m), m)$ and $A(\theta, m) = 0$, and take the Hermitian:

$$\frac{dJ(\theta(m), m)}{dm} = \frac{\partial J}{\partial \theta} \frac{d\theta}{dm} + \frac{\partial J}{\partial m} = -\lambda^* \frac{\partial A(\theta, m)}{\partial m} + \frac{\partial J}{\partial m}, \tag{15}$$

$$\left(\frac{\partial A(\theta, m)}{\partial \theta} \right)^* \lambda = \frac{\partial J}{\partial \theta}^* . \tag{16}$$

Since the partial derivatives are simple to compute, the problem lies in computing the total derivatives. Fortunately, this last step can be avoided by first solving equation 16 for λ, and then computing the final gradient via equation 15. Notice here that

the adjoint operator $(\partial A/\partial\theta)^*$ corresponds to the linearisation of the PDE A about the solution θ. Equation 16 is therefore very similar to the forward PDE system, with the difference that the "flow" of information is reversed by the transpose, plus a functional-dependant source term. In other words, computing the gradient via the adjoint method only requires solving a linear problem akin to that of the forward model. However, expressing the adjoint operator mathematically can be complex. Here we use the dolfin-ajoint module within FEniCS to: 1) automatically derive the adjoint code from the forward problem[6], and 2) perform the gradient descent step using the quasi-Newtonian L-BFGS-B algorithm[7].

Multi-scale analysis and large displacements

When displacements between consecutive images are too large (e.g. typically when the imaging frame rate is insufficiently high compared to intracellular movements), the assumption that displacements are small and local (cf. Equation 3) is no longer valid. To handle these situations, a multi-scale strategy is used[8]: a coarse-to-fine pyramid of sequences is obtained via iterative Gaussian filtering until a sufficiently coarse scale is reached (i.e. where the largest observable displacement is no more than one pixel). The displacements are first evaluated at the coarsest scale ($\overrightarrow{dx}_{pre}$) and then propagated to the next scale by: 1) warping the image to a new grid with third-order spline interpolation (I_{post}) and 2) adjusting the data attachment term J_{data} accordingly:

$$J_{\text{data}} = \int_{\Omega} \left(\nabla I_{post} \cdot (\overrightarrow{dx} - \overrightarrow{dx}_{pre}) + (I_{post} - I_1) \right)^2 d\Omega, \tag{17}$$

where the gradient operator is implemented as a five-point difference stencil (fourth-order accuracy). This two-step process is then iterated until the finest scale is processed, and the resulting method is capable of handling arbitrarily large displacements.

Automatic parameter estimation

The result and quality of the estimated variables depend on the choice of the empirical weighting factors in equations 9 and 12. Small values of α, γ, and η will tend to over-fit the difference between image pairs, including the experimental noise, and may favour the estimation of large forces. Conversely, large values of α or η (e.g. compared to γ) will either constrain the estimated forces or disregard the out of plane motion, which might no longer describe the observed movements correctly. In order to adjust these parameters in an unbiased manner, we employ an automated strategy that selects, for a given image pair, the parameter set that best predicts the previous and following images. In practice, this defines an error measure to be minimised, which is known as the Average Data Constancy Error (ADCE)[9]:

$$ADCE = \sum \left(I_{i-1} - \hat{I}_{i-1}\right)^2 + \sum \left(I_{i+2} - \hat{I}_{i+2}\right)^2 \tag{18}$$

where $\hat{I}_{i-1}$ is estimated by propagating I_i backwards using $-\vec{u}\Delta t$ via third-order spline interpolation, while I_{i+2} is obtained likewise by propagating I_{i+1} forward with $\vec{u}\Delta t$.

The minimum ADCE can be found using a fast derivative-free minimisation algorithm such as the Brent method[10]. Given that multiple parameters must be estimated simultaneously, we apply a two-step process, which we illustrate here for γ and α, regularising the force field. We first estimate γ and $\vec{g}$ using the unconstrained optical flow. Secondly, we run a second estimation for α and $\vec{f}$ with the constrained optical flow, keeping $\vec{g}$ constant. Thirdly, we fix the estimated α and γ and conduct a final joint estimation of $\vec{f}$ and $\vec{g}$ to refine the results.

Although the ADCE criterion is not guaranteed to be convex neither in α nor γ, we have found in practice that this criterion behaves well (its shape is close to parabolic) when either parameter varies on a logarithmic scale between reasonable bounds (see Supplementary Figure S3). In our experiments, we noted a range for α where the ADCE reaches a local plateau, indicating that any value within this range is equally satisfying. This behaviour is directly linked to the definition of the Stokes system (Equation 6). Indeed, the images are linked by $\vec{u}$, which is the first term of the first Stokes equation. There are however two possible ways to compensate $\mu\Delta\vec{u}$ for equality (either via ∇p or via $\vec{f}$), and α defines the balance between the two. We assume here that the cell favours small interior forces to minimise energy consumption (letting the pressure gradient become comparatively large[11]), and therefore select the largest value of α on that plateau to favour a large pressure gradient and small internal forces.

References

1. Gunzberger, M. D. *Perspectives in Flow Control and Optimization (Advances in Design and Control)* (2003).
2. Brezzi, F. On the existence, uniqueness and approximation of saddle-point problems arising from lagrangian multipliers. *ESAIM: Math. Model. Numer. Analysis* **8**, 129–51 (1974).
3. Girault, V. & Raviart, P.-A. *Finite Element Methods for Navier-Stokes Equations: theory and algorithms* (Springer, 1986).
4. Taylor, C. & Hood, P. A numerical solution of the Navier-Stokes equations using the finite element technique. *Comput. & Fluids* **1**, 73–100 (1973). DOI 10.1016/0045-7930(73)90027-3.
5. Talagrand, O. & Courtier, P. Variational Assimilation of Meteorological Observations With the Adjoint Vorticity Equation. I: Theory. *Q. J. Royal Meteorol. Soc.* **113**, 1311–1328 (1987). URL . DOI 10.1002/qj.49711347812.
6. Farrell, P., Ham, D., Funke, S. & Rognes, M. Automated derivation of the adjoint of high-level transient finite element programs. *SIAM J. on Sci. Comput.* **35**, C369–C393 (2013). URL . DOI 10.1137/120873558.
7. Byrd, R. H., Lu, P., Nocedal, J. & Zhu, C. A Limited Memory Algorithm for Bound Constrained Optimization. *SIAM J. on Sci. Comput.* **16**, 1190–1208 (1995). URL . DOI 10.1137/0916069.
8. Papenberg, N., Bruhn, A., Brox, T., Didas, S. & Weickert, J. Highly Accurate Optic Flow Computation with Theoretically Justified Warping. *Int. J. Comput. Vis.* **67**, 141–158 (2006).
9. Zimmer, H., Bruhn, A. & Weickert, J. Optic flow in harmony. *Int. J. Comput. Vis.* **93**, 368–388 (2011). DOI 10.1007/s11263-011-0422-6.
10. Brent, R. P. *Algorithms for Minimization Without Derivatives* (Dover Books, 2013).
11. Maugis, B. *et al.* Dynamic instability of the intracellular pressure drives bleb-based motility. *J. cell science* **123**, 3884–92 (2010). URL . DOI 10.1242/jcs.065672.

Supplementary Figures

Figure S1. Estimation of out-of-plane flow in 2D (a) Two consecutive frames of a 2D time-lapse microscopy sequence displaying out-of-plane motion (see Supplementary Movie S10); Scale bar: $10\mu m$. (b-c) Cell contours extracted from the first (black) and second (grey) frames, and streamlines of the extracted velocity field without (a) and with (b) estimation of out-of-plane flow. (d) Estimated out-of-plane flow r (s^{-1}); negative (resp. positive) values correspond to material leaving (resp. entering) the plane.

Figure S2. BioFlow at full resolution High (single-pixel) resolution maps of the intracellular forces (a) and velocity (b) presented in Figure 1(c) with equivalent colour scale (NB: arrows are not scaled according to magnitude for better visualisation).

Figure S3. Automatic parameter estimation Illustration of the dependency of the ADCE error measure on the empirical model parameters α and γ (see equations 9 and 12). The optimal parameter values are the minimum of each curve.

Movie S4. Video sequence of labelled *Entamoeba histolytica* Spinning-disk movie of a freely moving *Entamoeba histolytica* . The parasite is labelled with fluorescent Cythocalasin D (see *Methods*) and is analysed in Figures 2 and 3.

Movie S5. Bioflow Application of Bioflow to Movie S4. The pressue, forces and velocity fields are rendered on top of the original sequence with Icy.

Movie S6. 2D sequence Two consecutive 2D images extracted from Movie S4 and representing the sequence in Figure 1(a).

Movie S7. 3D sequence Two consecutive 3D images representing the sequence in Figure 1(d).

Movie S8. Other examples of BioFlow Examples of BioFlow on two more *Entamoeba histolytica* parasites.

Movie S9. Effect of Latranculin B Effect of Latranculin B on an *Entamoeba histolytica* parasite.

Movie S10. Out-of-plane flow Two consecutive 2D images displaying significant out-of-plane flow and representing the sequence in Figure 1(a).

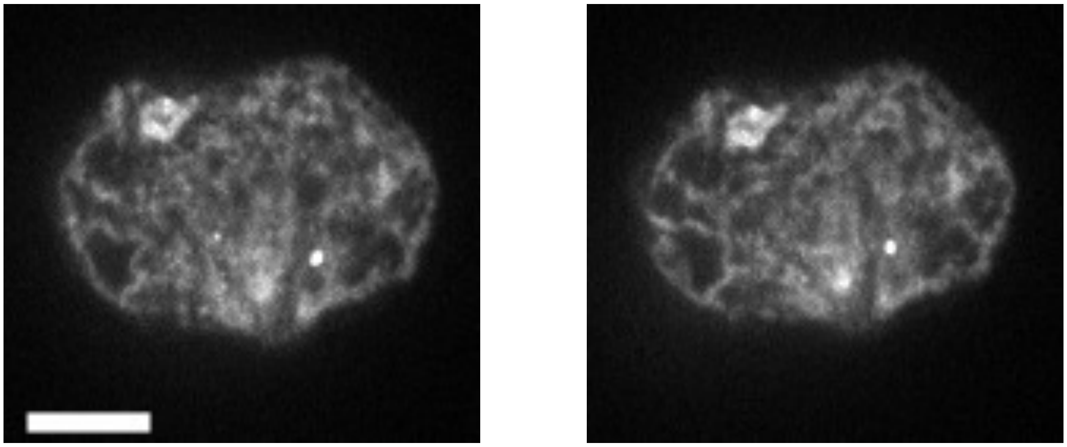

(a)

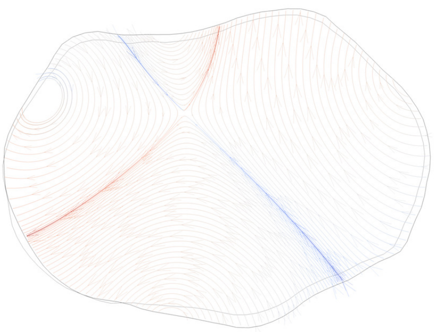

(b)

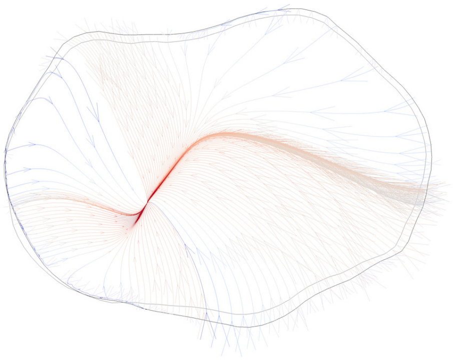

(c)

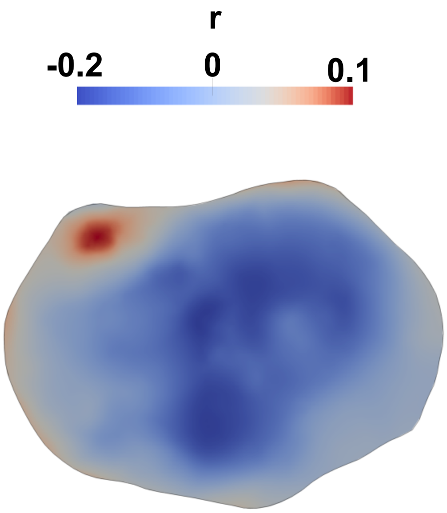

(d)

Supplementary Figure S1. **Estimation of out-of-plane flow in 2D.**

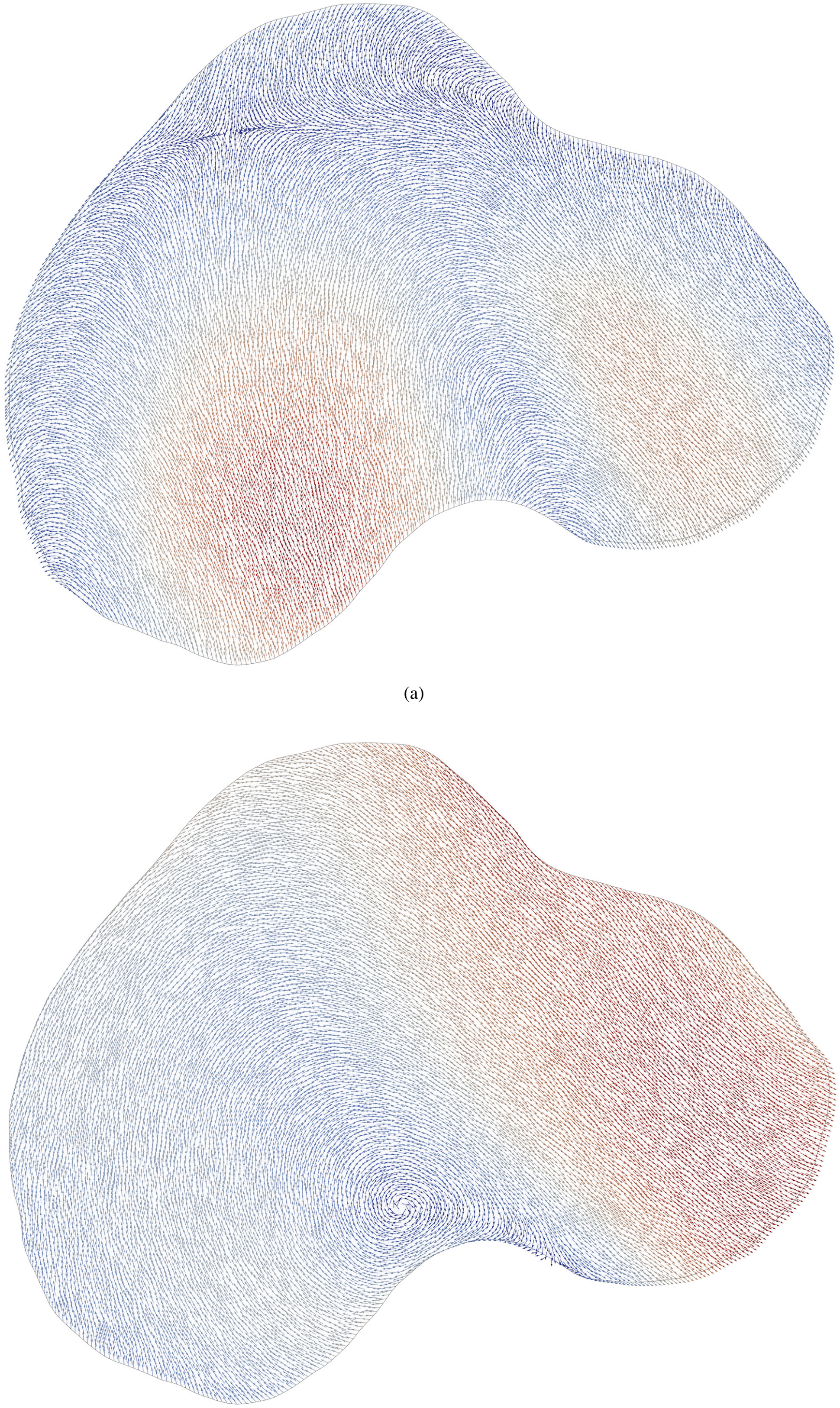

(a)

(b)

Supplementary Figure S2. **BioFlow at full resolution.**

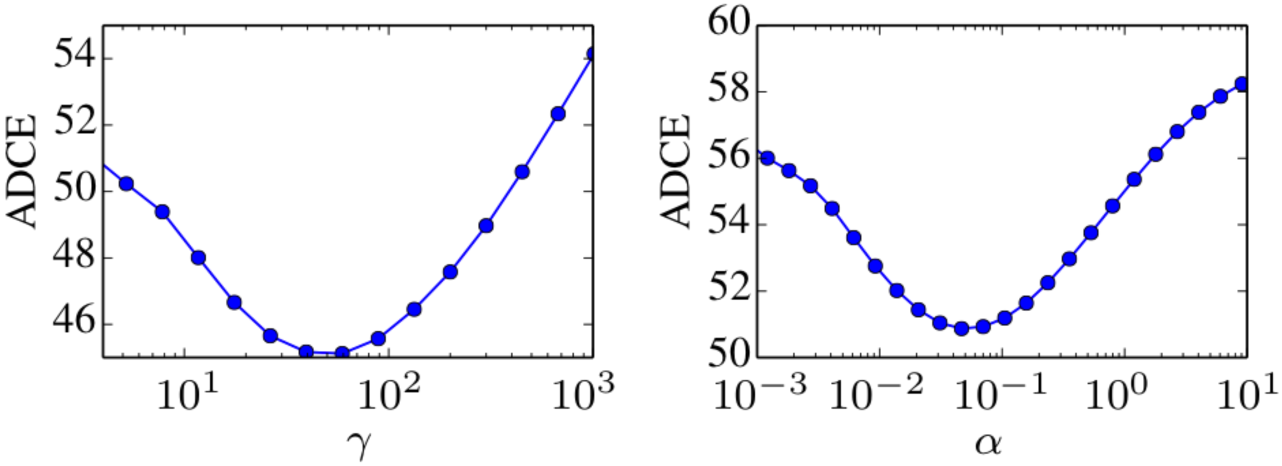

Supplementary Figure S3. **Automatic parameter estimation.**
